# Determining a target SpO_2_ to maintain PaO_2_ within a physiological range

**DOI:** 10.1371/journal.pone.0250740

**Published:** 2021-05-13

**Authors:** Jantine G. Röttgering, Angelique M. E. de Man, Thomas C. Schuurs, Evert-Jan Wils, Johannes M. Daniels, Joost G. van den Aardweg, Armand R. J. Girbes, Yvo M. Smulders

**Affiliations:** 1 Department of Intensive Care, Amsterdam UMC, Amsterdam, Noord-Holland, The Netherlands; 2 Institute for Cardiovascular Research (ICaR-VU), Amsterdam UMC, Amsterdam, Noord-Holland, The Netherlands; 3 Department of Emergency Medicine, Amsterdam UMC, Amsterdam, Noord-Holland, The Netherlands; 4 Department of Intensive Care, Franciscus Gasthuis & Vlietland, Rotterdam, Zuid-Holland, The Netherlands; 5 Department of Pulmonary Medicine, Amsterdam UMC, Amsterdam, Noord-Holland, The Netherlands; 6 Department of Internal Medicine, Amsterdam UMC, Amsterdam, Noord-Holland, The Netherlands; Heidelberg University Hospital, GERMANY

## Abstract

**Objective:**

In the context of an ongoing debate on the potential risks of hypoxemia and hyperoxemia, it seems prudent to maintain the partial arterial oxygen pressure (PaO_2_) in a physiological range during administration of supplemental oxygen. The PaO_2_ and peripheral oxygen saturation (SpO_2_) are closely related and both are used to monitor oxygenation status. However, SpO_2_ values cannot be used as an exact substitute for PaO_2_. The aim of this study in acutely ill and stable patients was to determine at which SpO_2_ level PaO_2_ is more or less certain to be in the physiological range.

**Methods:**

This is an observational study prospectively collecting data pairs of PaO_2_ and SpO_2_ values in patients admitted to the emergency room or intensive care unit (Prospective Inpatient Acutely ill cohort; PIA cohort). A second cohort of retrospective data of patients who underwent pulmonary function testing was also included (Retrospective Outpatient Pulmonary cohort; ROP cohort). Arterial hypoxemia was defined as PaO_2_ < 60 mmHg and hyperoxemia as PaO_2_ > 125 mmHg. The SpO_2_ cut-off values with the lowest risk of hypoxemia and hyperoxemia were determined as the 95th percentile of the observed SpO_2_ values corresponding with the observed hypoxemic and hyperoxemic PaO_2_ values.

**Results:**

220 data pairs were collected in the PIA cohort. 95% of hypoxemic PaO_2_ measurements occurred in patients with an SpO_2_ below 94%, and 95% of hyperoxemic PaO_2_ measurements occurred in patients with an SpO_2_ above 96%. Additionally in the 1379 data pairs of the ROP cohort, 95% of hypoxemic PaO_2_ measurements occurred in patients with an SpO_2_ below 93%.

**Conclusion:**

The SpO_2_ level marking an increased risk of arterial hypoxemia is not substantially different in acutely ill versus stable patients. In acutely ill patients receiving supplemental oxygen an SpO_2_ target of 95% maximizes the likelihood of maintaining PaO_2_ in the physiological range.

## Introduction

The administration of oxygen in acutely ill patients is one of the most frequently used therapeutic interventions, but the optimal target of the partial arterial oxygen pressure (PaO_2_) and peripheral oxygen saturation (SpO_2_) remains a topic of ongoing debate [1–3]. Some studies suggest that supra-physiological levels of PaO_2_, commonly referred to as hyperoxemia, may increase morbidity and mortality in critically ill patients [4–7], whilst for example a study in patients after cardiac arrest showed no negative effects of hyperoxia on mortality [8].The risk of hypoxemia has long been acknowledged, which commonly results in very liberal oxygen administration to acutely ill patients. Recent large randomized controlled trials in mechanically ventilated patients, however, showed no difference in mortality or other clinical outcomes between a mild hypoxemic and normoxemic protocol of oxygen therapy [9–12].

Non-invasive measurement of SpO_2_ by pulse oximetry is a derivative of arterial oxygen saturation (SaO_2_) and is used as a rapid and easy way to assess oxygenation. PaO_2_ on the other hand is the parameter of primary interest, since it reflects the balance between oxygen delivery and consumption [13, 14]. SaO_2_ and PaO_2_ are closely related, the nature of which is reflected by the oxyhemoglobin dissociation curve [15]. However SaO_2_ or SpO_2_ cannot be interpreted as an exact substitute of PaO_2_ [16, 17]. Lower hemoglobin levels, acid–base disturbances and altered temperature all influence the correlation between SaO_2_ and PaO_2_ [18] and altered cardiac output, peripheral tissue perfusion, sepsis and the use of vasoactive medication disturb the accuracy of SpO_2_ measurements [19–24]. Also, the measurement of saturation is intrinsically limited in that it cannot detect hyperoxemia if SpO_2_ is close to or at its maximum value of 100%. Indeed, corresponding PaO_2_ values for any given SpO_2_ value may range widely [24–27].

In order to prevent hypoxemia and hyperoxemia and its possible associated risks, the British Thoracic Society Guideline recommends a target SpO_2_ of 94–98% in adults in healthcare and emergency settings [28]. Others guidelines, however, caution against administering oxygen to obtain an SpO_2_ above 96% and advocate a lower SpO_2_ range [29, 30]. A recent pilot study by our group in a small cohort of Intensive Care Unit (ICU) patients determined that the risk of arterial hyperoxemia (> 125 mmHg), was negligible when SpO_2_ was 96% or lower [31].

In light of the debate on the trade-off between the risks of arterial hypoxemia and hyperoxemia, it is important to increase our understanding of the SpO_2_–PaO_2_ relationship, particularly in acutely ill patients receiving oxygen. In this study, we aim to identify a target SpO_2_ value, with relative safety margins, resulting in a high likelihood of maintaining PaO_2_ within a physiological range while administering oxygen [32].

## Materials and methods

### Study design

We performed a prospective observational study collecting simultaneously measured PaO_2_ and SpO_2_ values in two different patient cohorts. The study was registered at clinicaltrials.gov (NCT02666937). This cohort consists of acutely ill patients admitted to the Emergency Room (ER) and the ICU and is referred to as the Prospective Inpatient Acutely ill cohort (PIA cohort).

The medical research ethics committee of the Amsterdam University Medical Centers (Amsterdam UMC) approved the study protocol. The patients in the PIA cohort were informed about the use of their data and could object to their data being used through an opt-out procedure. The medical research ethics committee specifically approved the use of opt-out consent for these patients.

We also included a second cohort of patients with simultaneously measured PaO_2_ and SpO_2_ values. This was a retrospective dataset of patients undergoing pulmonary function testing. This dataset was used to gain more insight into the lower range of SpO_2_ and PaO_2_ values in a larger population. This cohort of patients is referred to as the Retrospective Outpatients Pulmonary cohort (ROP cohort). The medical research ethics committee granted a waiver of consent for the use of this data.

### The Prospective Inpatient Acutely Ill cohort (PIA cohort)

For the PIA cohort, data was collected prospectively from December 2015 to August 2017 in mechanically ventilated ICU-patients and acutely ill patients visiting the ER at the Amsterdam UMC. This cohort consisted of a convenience sample, based on the availability of researchers. Adult patients were eligible for inclusion if they required arterial blood gas analysis (ABGA) as part of routine care. Up to 10 data pairs could be collected per patient with a time interval of at least two hours between samples. A data pair is one single measurement of a combined SpO_2_ and PaO_2_ value.

Simultaneous with the ABGA the SpO_2_ was measured with the use of a Nellcor OxiMax DS-100A (Covidien, Mansfield, USA) pulse oximeter. Reliability of the SpO_2_ curve was assessed and defined as a curve showing complete pulsatile tracing of the beat-to-beat arterial pulse wave on the pulse oximeter. On the ICU, PaO_2_ was measured using a combined blood gas analyzer and CO-oximeter (ABL825, pH-stat, Radiometer, Copenhagen, Denmark). The samples were analyzed immediately after sampling.

Patients with methemoglobinemia (> 1.5%), carboxyhemoglobinemia (> 1.5%) or known hyperbilirubinemia (> 20 μmol/L) were excluded. Additionally, ICU patients were only included if they had an arterial cannula and required mechanical ventilation, and were excluded when undergoing extracorporeal membrane oxygenation or therapeutic hypothermia. Oxygen was titrated to achieve a stable arterial oxygen saturation of ≥ 92% in the ICU during routine clinical care. ER patients were included upon presentation when they required ABGA. Most ER patients were stabilized with a non-rebreather mask with 15 liter O_2_/min when at risk for insufficient oxygenation, according to local guidelines.

### The Retrospective Outpatient Pulmonary cohort (ROP cohort)

For the ROP cohort, a dataset was used containing adult patients performing a pulmonary function test at the Amsterdam UMC between 1995 and 2014 and at the Northwest Clinics between 2016 and 2017. A researcher accessed the database to obtain the retrospective data in July 2017. All patients having performed a test were screened for eligibility and were included if they had had an ABGA and a simultaneous SpO_2_ measurement. Per patient between one and four ABGA’s were collected during each pulmonary function test. The pulmonary function test consisted of a cycling protocol without supplemental oxygen. The arterial puncture was performed by a pulmonary function technician in the radial artery. SpO_2_ was measured with a NONIN Avant 9600 (Nonin Inc., Plymouth, MN) pulse oximeter at the Amsterdam UMC and a NONIN 8000S (Nonin Inc., Plymouth, MN) pulse oximeter at the Northwest Clinics.

### Defining hypoxemia and hyperoxemia

The exact cut-offs for hypoxemia and hyperoxemia are relatively arbitrary and not well defined in literature. For example hyperoxemia is defined in a range from > 120 mmHg [33] up to > 300 mmHg [34], although the latter is clearly far above the physiological value when breathing a normal air mixture. In a multinational survey, most ICU-doctors stated they would accept a PaO_2_ of 60 mmHg or lower in a clinical trial of oxygenation targets [35]. We therefore pragmatically defined hypoxemia as a PaO_2_ < 60 mmHg [33, 34, 36] and hyperoxemia as a PaO_2_ > 125 mmHg [31, 37, 38].

### Statistical analysis

Data were analyzed using SPSS (version 22, IBM, Armonk, NY, USA) and RStudio (version 3.5.3, R Foundation for Statistical Computing, Vienna, Austria). Values are presented as mean with standard deviation (SD) unless otherwise stated.

SpO_2_ and PaO_2_ data were first visually assessed with a scatterplot. We determined a hypoxemic and hyperoxemic SpO_2_ limit for the PIA cohort. The hypoxemic SpO_2_ limit was determined as the 95th percentile of the observed SpO_2_ values corresponding with the observed hypoxemic PaO_2_. The hyperoxemic SpO_2_ limit was determined as the 5th percentile of the observed SpO_2_ values corresponding with the hyperoxemic PaO_2_ values. For the ROP cohort we only defined a hypoxemic SpO_2_ limit, since the patients did not receive supplementary oxygen.

Since multiple measurements were available for most patients, we constructed a mixed linear model in both cohorts. This included a random effect for each patient. First SpO_2_ and PaO_2_ values were transformed such that relation between the SpO_2_ and PaO_2_ became linear (ln(1—SpO_2_/100) and ln(PaO_2_)). The transformed SpO_2_ was the dependent variable and the transformed PaO_2_ was included as a covariate with fixed effect. A two-sided 95% prediction interval was constructed for each specific value of PaO_2_. The upper and lower limit of the prediction interval and the predicted mean value for SpO_2_ were then transformed back to the original scale.

## Results

### Included patients and data pairs characteristics

**Fig 1:**
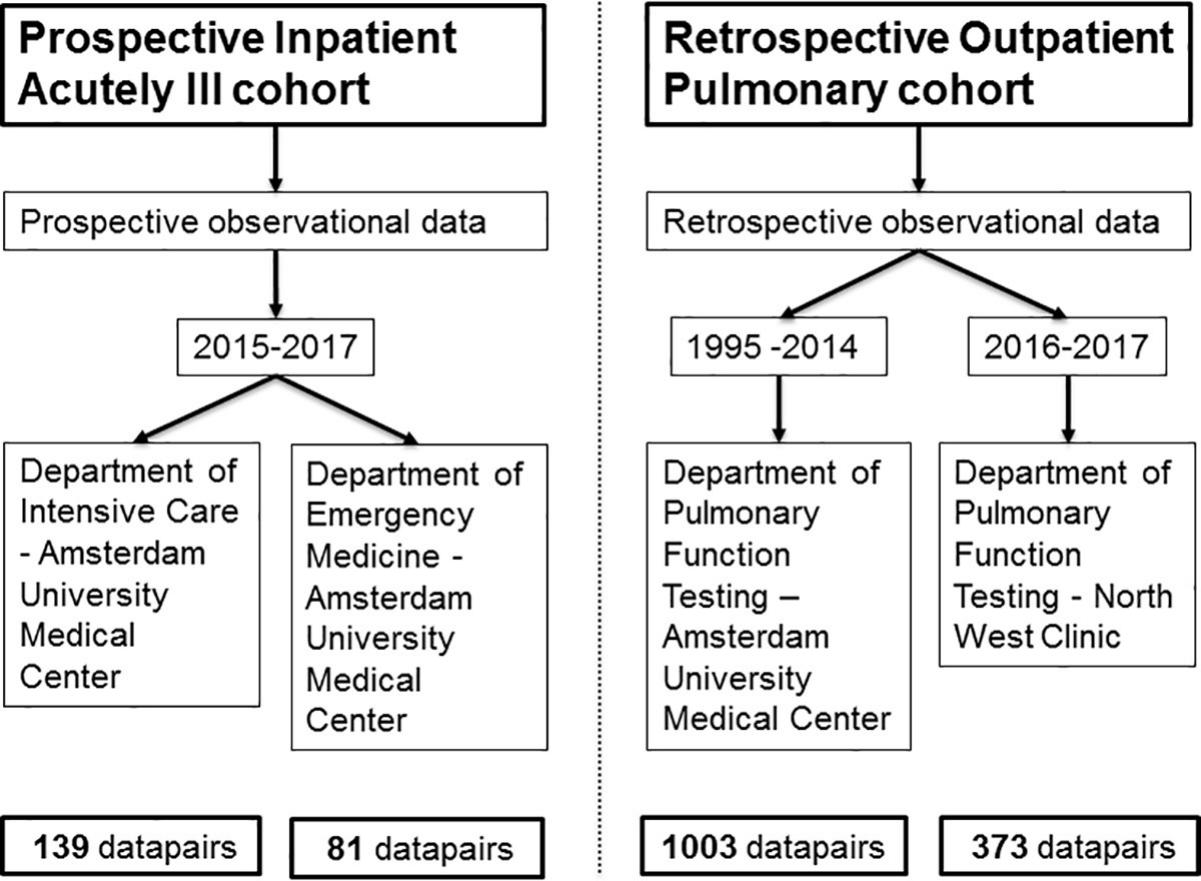
Visual representation of the included cohorts.

We screened 152 patients in the PIA cohort for eligibility. One patient was excluded because of hyperbilirubinemia and five were excluded because of unreliable SpO_2_ curves, resulting in the inclusion of 220 data pairs in 146 patients. 139 data pairs were collected at the ICU and 81 at the ER. We screened 693 patients in the retrospective database for eligibility in the ROP cohort. Thirteen patients were excluded because of no or unreliable SpO_2_ measurements, resulting in the inclusion of 1379 data pairs in 680 patients. 1003 data pairs were collected at the Amsterdam UMC, and 376 pairs at the North West Clinic. Fig 1 shows a visual representation of the different cohorts.

Demographics of the included patients and characteristics of the collected data pairs are shown in Table 1. In the PIA cohort, 6.4% of the data pairs had a hypoxemic PaO_2_ < 60 mmHg, and 20.5% had a PaO_2_ > 125 mmHg. In the ROP cohort 10.7% of the data pairs had a PaO_2_ value < 60 mmHg.

**Table 1:** Demographics of included patients and data pairs characteristics.

	Prospective Inpatient Acutely Ill (PIA) cohort			Retrospective Outpatient Pulmonary (ROP) cohort		
**Demographics patients**			**N = 146**			**N = 680**
Age in years	63	(17)		58	(15)	
Male	96	66%		362	53%	
Intensive Care Unit Amsterdam UMC	68	47%		-		
Emergency Room Amsterdam UMC	78	53%		-		
Pulmonary function department NWC	-			68	47%	
Pulmonary function department Amsterdam UMC	-			78	53%	
**Collected data pairs**			**N =**			**N =**
SpO_2_ (%)	96.6	(4.6)	220	94.7	(4.4)	1379
SaO_2_ (%)	96.1	(4.5)	212	95.4	(5.5)	1357
PaO_2_ (mmHg)	110.9	(64.6)	220	88.6	(20.6)	1379
PaO_2_ < 60 mmHg	14	6.4%	220	148	10.7%	1379
PaO_2_ > 125 mmHg	45	20.5%	220	31	2.2%	1379
PaCO_2_ (mmHg)	43.6	(13.2)	220	36.8	(6.1)	1377
pH	7.36	(0.13)	218	7.37	(0.06)	1378
Arterial Bicarbonate (mmol/l)	24.1	(5.3)	216	21.7	(3.8)	1359
Arterial Lactate (mmol/l)	2.37	(2.9)	210			
Hemoglobin (mmol/l)	6.9	(1.4)	183			
Systolic blood pressure (mmHg)	127	(28)	214	179	(38)	1043
Diastolic blood pressure (mmHg)	68	(17)	214	88	(16)	1041
Supplemental oxygen (%)	191	87%	219	0	0%	1379

Data are presented as a mean (standard deviation) or number with % of total.

Amsterdam UMC, Amsterdam University Medical Center; N, number of patients; NWC, North West Clinic; PaCO_2_, partial arterial carbon dioxide pressure; PaO_2_, partial arterial oxygen pressure; SaO_2_, peripheral oxygen saturation; SpO2, peripheral oxygen saturation

### Relationship between SpO_2_ and PaO_2_

**Fig 2:**
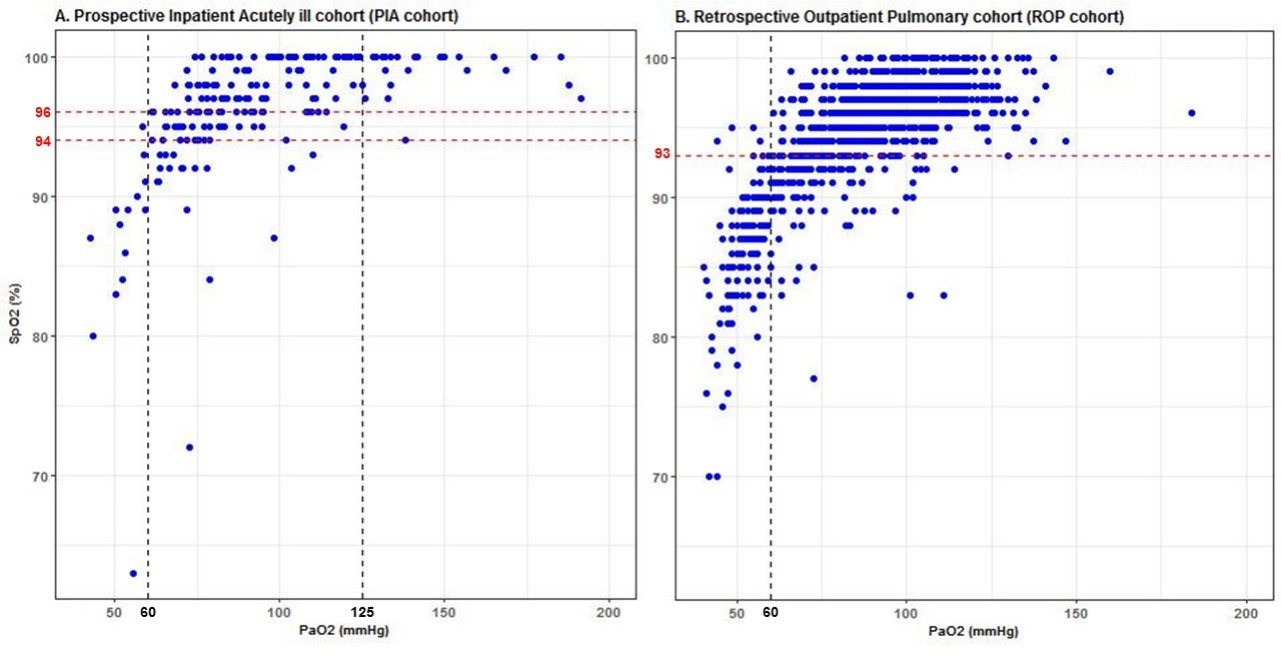
Scatterplots of the data pairs of the Prospective Inpatient Acutely ill cohorts and Retrospective Outpatient Pulmonary cohort. A. Prospective Inpatient Acutely ill cohort (PIA cohort) and B. Retrospective Outpatient Pulmonary cohort (ROP cohort). The black dashed vertical lines reflect the PaO_2_ values of 60 mmHg and 125 mmHg. The red dashed vertical lines reflect the calculated lower and upper bounds to avoid hypoxemic and hyperoxemic PaO_2_ measurements.

Fig 2 shows the scatterplots of the data pairs of PaO_2_ and SpO_2_ measurements for both cohorts. In the PIA cohort 95% of data pairs corresponding to hypoxemia had SpO_2_ values below 94%, and 95% of data pairs corresponding to hyperoxemia had SpO_2_ values exceeding 96%. The horizontal red dotted lines in Fig 2 reflect the hypoxemic and hyperoxemic SpO_2_ limits. The patients in the ROP cohort have more hypoxemic PaO_2_ values and did not receive supplemental oxygen. In the ROP cohort, 95% of data pairs corresponding to a hypoxemic PaO_2_ had SpO_2_ values below 93%.

**Fig 3:**
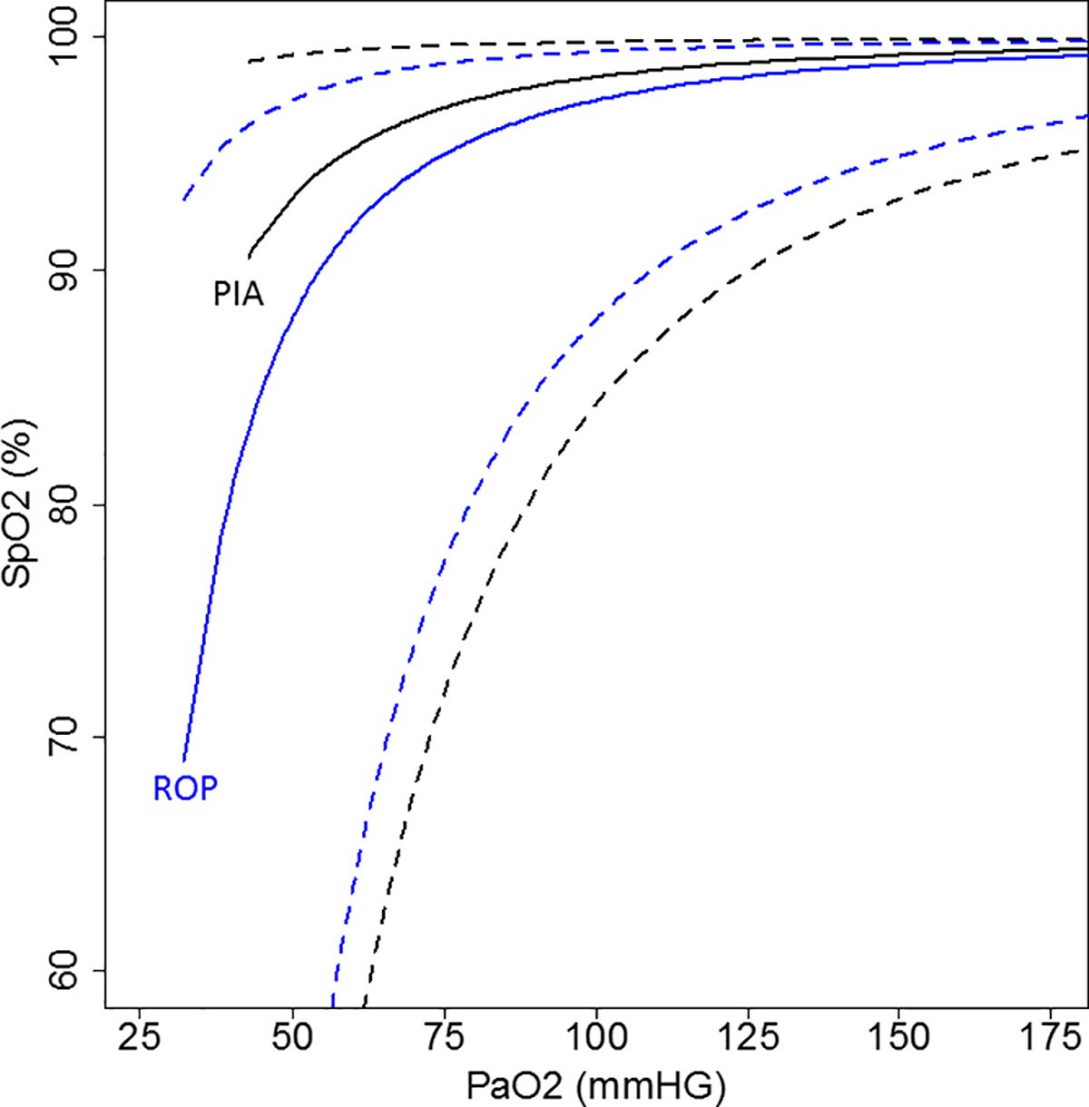
Multilevel analysis of the Prospective Inpatient Acutely ill and Retrospective Outpatient Pulmonary cohort. PIA, Prospective Inpatient Acutely ill cohort. ROP, Retrospective Outpatient Pulmonary cohort. The black curve represents the Prospective Inpatient Acutely Ill cohort with a 95% prediction interval (black dashed line). The blue curve represents the Retrospective Outpatient Pulmonary cohort with a 95% prediction interval (blue dashed line).

A multilevel analysis was performed for each cohort, taking into account that multiple measurements could be included per patients (see Fig 3). The curve of the PIA cohort runs slightly left of the ROP curve. There are more measurements with a PaO_2_ >125 mmHg in the PIA cohort and more measurements with a PaO_2_ < 60 mmHg in the ROP cohort

**Fig 4:**
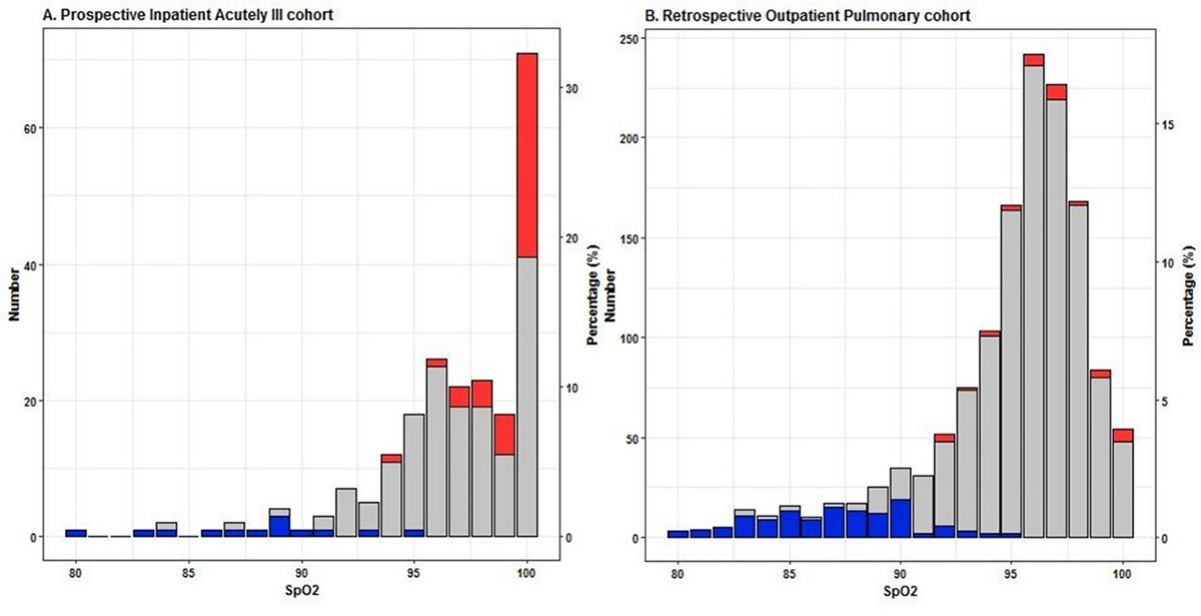
Stacked bar plots of the number and percentage of PaO_2_ values at every SpO_2_ value. A. Prospective Inpatient Acutely ill cohort (PIA cohort) and B. Retrospective Outpatient Pulmonary cohort (ROP cohort). The left y-axis represents the number of data points and the right y-axis represents the percentage of data points per SpO_2_ value of the total number of measurements in the whole cohort. PaO_2_ values are divided into three groups: < 60 mmHg (blue), 60–125 mmHg (gray) and > 125 mmHg (red).

Fig 4 shows the number of PaO_2_ measurements within a certain PaO_2_ range for each SpO_2_ level in both cohorts. In the PIA cohort at an SpO_2_ value of 95%, 94,4% of the data pairs had a PaO_2_ value between 60 and 125 mmHg. For an SpO_2_ value of 96% and 98%, the corresponding rates of hyperoxemic values were 3.8% and 17.4%, respectively. In the ROP cohort at an SpO_2_ value of 93%, 4% of the data pairs had a hypoxemic PaO_2_ value. For an SpO_2_ value of 92%, the corresponding rate was 11.5%.

## Discussion

In the PIA cohort with SpO_2_ values of 94–96% both hypoxemia and hyperoxemia can be effectively excluded. In the ROP cohort hypoxemia can be excluded with a lower SpO_2_ target of 93%. The lower PIA and ROP targets excluding hypoxemia are not substantially different. Combining these cut-offs, we recommend an SpO_2_ target of 95%, which clearly has margins of relative safety. This target SpO_2_ results in the highest likelihood of physiological PaO_2_ values for the average patient.

Our findings are supported by data from an uncontrolled study, aiming at a target SpO_2_ range between 92–95% that indeed resulted in a significant decrease of the number of hyperoxemic episodes, while hypoxemic episodes remained unchanged [39]. Moreover, an observational study in mechanically ventilated ICU patients by our group also suggested a safe upper SpO_2_ level excluding hyperoxia of 96% [31]. As for safety in terms of clinical outcomes, a recent systematic review of randomised studies in acutely ill patients showed that, in a variety of acute clinical conditions, mortality increased in patients with an SpO_2_ levels above 96% [29]. The British Thoracic Society Guideline (2017) recommends more liberal use of oxygen and an upper limit for SpO_2_ of 98% for acutely ill patients [28]. Our data suggest that this recommended SpO_2_ target of 98% may result in more hyperoxemia, without being associated with a meaningful lower risk of hypoxemia.

In the ROP cohort an SpO_2_ value below 93% results in a higher risk of hypoxemia, for the PIA cohort this is the case below 94%. The lower limits are quite similar in these different cohorts of acutely ill and stable patients. These lower safe limits are in line with recent clinical guidelines recommending a lower SpO_2_ limit of 92–94% [28, 29]. Aiming for even lower SpO_2_ values increases the risk for hypoxemia.

This observational study has some limitations. Different oximeters were used for the two cohorts and potential differences between measurements can depend on the oximeter used. The Nellcor oximeters, used in the PIA cohort, slightly overestimate SaO_2_ [40, 41], while Nonin oximeters, used in the ROP cohort, might underestimate SaO_2_ [41]. Since the lower limit in the PIA cohort was possibly overestimated and the lower limit in the ROP cohort was possibly underestimated, the lower limit for the two cohorts are probably the same. This strengthens the assumption that the hypoxemic safe margin of SpO_2_ is not heavily influenced by specific pulmonary morbidity.

It is important to note, that there is no clear definition of hypoxemia and hyperoxemia available. We therefore pragmatically defined hypoxemia in line with literature and clinical practice as PaO_2_ < 60 mmHg [33, 34, 36] and hyperoxemia as PaO_2_ > 125 mmHg [31, 37, 38]. Different definitions would have resulted in different outcomes of this study. It should be noted that the potential risks of hyperoxemia remain unclear. Some studies do [4–7] and some studies do not [8] show an effect of hyperoxemia on mortality. However in view of the controversy on the definitions and clinical hazards of hypoxemia and hyperoxemia, it seems prudent to maintain PaO_2_ in a physiological range, defined as between 60 and 125 mmHg [32].

The PIA cohort consisted of a convenience sample. The patient profiles in the PIA cohort varied, with mechanically ventilated patients at the ICU and patients requiring blood gas analysis at the ER. In both cohorts, the number of patients with hypoxemia was relatively small, because of oxygen administration. The ROP cohort consisted of retrospective data of patients performing a pulmonary function test with a cycling protocol. There are very few alternatives to the ROP cohort to obtain more insight in hypoxemic PaO_2_ values, since stable patients without pulmonary pathology will hardly ever be hypoxemic. Thus, this limitation is intrinsic to the study question of whether the safe SpO_2_ margin is different in acutely ill versus stable patients.

This study did not explore the clinical variables potentially modifying the relation between PaO_2_ and SaO_2_ or the accuracy of SpO_2_ measurements, so we cannot provide separate recommendations about specific conditions, such as sepsis, hypotension, heart failure, or acute respiratory failure. The generalizability of the results of this study should be confirmed with sufficiently powered studies in patients with specific conditions with different oximeters to define how clinical variables might change SpO_2_ targets. Also the different definitions of hypoxemia and hyperoxemia and clinical outcome variables should be taken into account in future studies.

In conclusion, we recommend an SpO_2_-target of 95%, or as close to this level as possible, to avoid the occurrence of either hypoxemia or hyperoxemia in acutely ill patients receiving supplementary oxygen.

## Supporting information

S1 File (XLSX)

## Abbreviations

ABGA: Arterial blood gas analysis
Amsterdam UMC: Amsterdam University Medical Centres
ER: Emergency Room
ICU: Intensive Care Unit
NWC: Northwest Clinics
PaCO_2_: Partial arterial carbon dioxide pressure
PaO_2_: Partial arterial oxygen pressure
PFD: Pulmonary function department
PIA cohort: Prospective Inpatient Acutely Ill cohort
ROP cohort: Retrospective Outpatient Pulmonary cohort
SaO_2_: Arterial oxygen saturation
SBP: Systolic blood pressure
SD: Standard deviation
SpO_2_: Peripheral oxygen saturation
